# Some Observations on the Heart

**Published:** 1916-05

**Authors:** M. T. Davis

**Affiliations:** 931 Candler Bldg., Atlanta, Ga.


### SOME OBSERVATIONS ON THE HEART.

By M. T. Davis, M. D.

We who sit at the feet of those older in the profession, and therefore wiser than ourselves, and drink at the fount of their knowledge, born of expeience, are loth to advance any new ideas of our own, lest we be treading on hallowed ground, and amid fields which investigation will disclose have already been explored.

Disclaiming the intention or desire to essay such a task, I shall invite your attention only to some of the frequently forgotten or overlooked points touching cardiac conditions generally.

The fact that a murmur is heard about the heart is no sign of itself that the heart is disused at all, since there are many murmurs that are independent of actual cardiac disease, however closely they may imitate true organic murmurs. That is, we may have Hemic, Neurotic, Intra-arterial and cardio-respiratory murmurs, as well as pleurocardial and pericardial friction sounds. It is our duty by careful, intelligent observation, to distinguish between these, since the diagnosis, prognosis and treatment will depend upon our findings.

Without a fairly accurate knowledge of the anatomy and physiology of the heart, and a perfectly clear conception of what is taking place during every moment of the cardiac cycle, it is difficult to understand valvular diseases of the heart with their resultant hypertrophy; and it is impossible to comprehend the principle of murmurs with this time of occurrence, points of maximum intensity, and areas of transmission or conduction.

If my remarks shall seem elementary to those who are constantly studying the heart, I hope I may be pardoned, believing that they may prove of interest to others of us who may have grown a bit rusty on a subject with which we were once famaliar.

Probably in every acute systemic infection the heart suffers to a greater or less degree, and only recovers with a scar left upon its musculature, or upon its peri or endocardium. These scars throughout the future, spell weakness, or strength.

In some of the acute infections, especially diphtheria, the pulse becomes thready, cyanosis, profuse sweating, nausea and vomiting supervene, the blood pressure falls, and the patient dies with all the phenomena of shock. Such a death results primarily from damage to the structural mechanism of the heart, mainly its myocardium and is due to the toxins circulating in the blood. In the event of recovery, the heart often fails to regain its full integrity, and dates its first clinical insufficiency from the acute systemic infection. The picture is too often classed as the debility of convalescence, or as a post-febrile anemic state. The important point is to recognize that there IS a real loss, and lack of ability to maintain cardiac muscular tone. We should watch intelligently from the beginning for the early signs of cardiac inability and later for indications of cardiac distress. A weak, irregular pulse, air-hunger, precordial oppression, and pain that cannot be attributed to pericarditis, will all have a new significance when we realize that the MAIN lesion, even in Rheumatism, is of the myocardium, not of the valves, and is just as likely to be permanent as if it were valvular. Many of us have learned a severe clinical lession in the loss of a patient, owing to sudden cardiac failure, when the convalescence has seemed quite complete. Myocardial degeneration menaces, the health, and often the life, of every patient suffering from diphtheria, scarlet fever, syphilis, typhoid fever, the pneumonias, true influenza, rheumatism, tonsilitis and chorea.

If myocardial tone is lacking every emergency is a new burden. Nor must we forget that the arterial system is simply a continuation of the structural architecture of the heart, and that factors which lower cardiac muscular tone, are apt to have the same influence on the muscular fibres in the walls of the arteries and veins. Fibrous arteries in a YOUNG person, constitute presumptive evidence of syphilitic myocardial disease, with its resultant fibrosis or sclerosis ramifying throughout the arterial tree.

We are too prone to think that the dilated heart is incompetent as a result of misbehavior of some valve or valves, and to ignore the covering and musculature of the organ. We forget that the same infection may cause endocarditis, pericarditis and myocarditis. Let us be on the lookout for these in every case of acute systemic invasion by bacterial organisms.

The heart may at times be responsible for various nervous and mental manifestations of a reflex character. There are two symptoms referable to the nervous system which, while not common, are by no means rare in disturbed or broken compensation: First, a reflected pain along the distribution of the fifth nerve; and second, a mental and emotional abnormality, aside from any worry about the disease, characterized by depression, anxiety and fear, often with a sense of impending diseaster. These emotional states may occur with or without distinct delusions and hallucinations. Nightmare may be caused by irregularity of the heart's action, the patient waking from a distressing dream, often with a sense of oppression and with extreme bradycardia.

All heart patients should be closely questioned about their sleeping and dreaming, for it is quite possible that periods of failing compensation might be forestalled in certain instances, if rest were instituted at the first appearance of these marked nervous phenomena.

The neuralgia of the fifth nerve, which I have referred to, and the scalp tenderness over the area of its distribution, are so common, that in chronic valvular cases the onset of neuralgia may precede a period of failing compensation so regularly that the patient himself learns that the pain is a signal that he must rest.

That peculiar retrograde tissue metamorphosis, known as fatty degeneration of the heart, is a form of molecular death often due to impaired nutrition of the heart muscle. It is most often seen in hearts that are hypertrophied. Now, a common cause of hypertrophy is high blood pressure in the arteries, with a sclerotic condition, and a diminution of their elasticity. Their recoil, after distension by the ventricular systole is impaired. This recoil in the aorta is who pels the blood into the coronary arteries. When it is impaired, the pressure of blood in the coronary arteries is lessened, and the blood suppy to the heart muscle itself is diminished, with a resultant impaired nutrition of the heart. The hypertrophied heart, no longer sufficiently supplied with blood, commences to dilate, yielding to the distending force of the contained blood, resulting in failing hypertrophy, or what is NOW known AS BROKEN COMPENSATION.

Fatty degeneration is most often a disease of advanced life, and the nervous symptoms at times manifested, are both interesting and pathetic. Cerebral anemia is the result of impaired arterial fulness. The enfeebled heart can no longer maintain a full pressure in the cerebral arteries, and evidences of brain failure are manifested accordingly. Hence we should not be surprised at the querulousness, the caprice and the rapid variation of temper as the blood pressure in the cerebral vessels alters from time to time. The vascillating procrastination exhibited by these patients, their inability to decide on matters, their unreasonable and inexplicable conduct, their whims, preferences and dislikes, are ALL OF REAL DIAGNOSTIC VALUE

There may be acute attacks of cerebral anemia not unlike syncope, or, at other times it may simulate apoplexy. The man with a hesitating heart is thereby unfitted for sudden tasks, demands, or resolves, for when the heart hesitates, the brain, which reposes for ITS POWER on the blood the heart supplies it, falters WITH the heart, just as the gas flickers when the steady pressure is taken off the main. Hence, we should bear very patiently with elderly people who were once resolute and determined, but who have now lost these qualities, and become uncertain and doubtful in character, themselves knowing that they no longer have self-mastery. We should allow for their enfeebled mental processes, and bear with what appears to be stupidity, for these old people may have fatty failing hearts, and IF THE TRUE ARCUS SENILIS IS PRESENT, NEVER FAIL to at least SUSPECT IT.

How often does the general practitioner overlook the heart in treating diseases of the stomach! Indeed it now and then happens, as occurred in my own case, that a patient comes to us right from the hands of the stomach specialist, who through oversight has neglected to make a careful examination of the heart. No amount of dieting, lavage, alkaline powders or hydrochloric acid and pepsin will avail anything in the presence of a relative tricuspid regurgitation, from enormous hypertrophy of th right ventricle as the result of mitral stenosis. Herce the blood is being dammed back throughout the venous system, the brunt of the burden falling upon the splanchnic area, and we have venous statis in the vessels [illegible] normally return the blood from the musculature and mucosa of the stomach. This stasis means a chronic state of congestion or engorgement which nothing will relieve until rest and large doses of digitalis have restored to the crippled right ventricle, sufficient power to enable it to empty itself completely at each systole, and thus make room for the blood dammed back in the veins of all the viscera. It sounds academic at this day to urge the necessity of having the patient strip to the waist for a heart examination. The sounds produced by even a thin towel again the ear or stethoscope, will obscure the finer points in auscultatory diagnosis, and yet how often do physicians listen to the chest with one or more garments intervening!

Within the past few years we have come to have a better understanding of what takes place within the heart in [illegible]se to certain stimuli, intrinsic and extrinsic. We know that the primary stimulus for contraction originates normally in the node of Keith and Flack, or the sinus node, the pace-maker of the heart which lies i nthe upper part of the right auricle, near where it joins the superior vena cava. If from any cause this region is disturbed, an impulse may come from some other part of the auricle, from the ventricle, or some point between the two, giving rise to what is known as EXTRASYSTÔLES.

(2) We know that there is a clinical entity often difficult of diagnosis, due to changes in the sinus node, charatceerized by marked irregularity of the heart of long duration, formerly called PULSUS IRREGULARIS PERPETUUS, but which is now known as AURICULAR FIBRILLATION. In this, the normal auriular systolic contractions do not occur, but in their stead, there are little rapid twitchings of the muscle fibres of the auricles, often several hundred to the minute, which gives the name to the condition. Fortunately the ventricle only responds to a certain number of these auricular stimuli, else it would soon wear itself out.

(3) We know that the auriculo-ventricular bundle of His, normally conducts the auricular impulse to the ventricle. If this function is interfered with, it causes the ventricle to drop one or more of its beats. This condition is known as HEART-BLOCK, and if intermittent only, constitutes the ADAMS-STOKES SYNDROME, with cerebral anemia pallor, syncope, etc.

We have also recently learned that so delicate is the balance of the heart mechanism, that for proper functioning, there must not only be perfection of muscle, nerve, and heart circulation, BUT THE SUBSTANCES IN SOLUTION IN THE BLOOD must be in proper amounts and relationship to each other, for heart stimuli to be normal. Viewed in this light it is not surprising that irregularities of the heart's action, or the so-called arhythmias may be engendered by myocarditis, broken compensation, coronary disease, the organic toxins poured into the blood stream during acute infections, tea and coffee, alcohol and tobacco. Nor must we ignore the role played by gastric indigestion and intestinal fermentation, with the absorption of toxic principles, in the production of disturbances of the heart hitherto ascribed to other causes. The field is a broad and inviting one, and affords opportunity not alone for speculation, but to-day for actual bio-chemic demonstration of the possibilities at which I have only hinted.

931 Candler Bldg., Atlanta, Ga.

